# Supplementary material for: Spatial and Temporal Genetic Diversity of the Peach Potato Aphid Myzus persicae (Sulzer) in Tunisia
Source: Insects. 2019 Oct 1;10(10):330. doi: 10.3390/insects10100330 (PMC6835240; doi:10.3390/insects10100330)
Supplement: Supplementary file 1 [file insects-10-00330-s001.pdf]

SUPPLEMENTARY MATERIAL

Table S1. Allele combinations for each of the 548 aphid individuals of *Myzus persicae* sampled in Tunisia from 2011 to 2016.

ID	Pop	Host	Locality	Saison	Date	Myz9	M37	M40	M49	M63	M86	Genotype	unique/multiple/population
1	pop1	potato	Cap-Bon	Spring	31/03/2016	212240	178180	146146	150150	188194	151151	G306	1
2	pop1	potato	Cap-Bon	Spring	31/03/2016	212224	176178	142142	156156	188194	149149	G187	1
4	pop1	potato	Cap-Bon	Spring	20/04/2016	212212	178178	136136	150150	194194	129129	G124	2
5	pop1	potato	Cap-Bon	Spring	20/04/2016	238240	172170	136142	168168	186194	125125	G388	4
6	pop1	potato	Cap-Bon	Spring	20/04/2016	224240	178180	142148	154154	188188	129149	G334	1
7	pop1	potato	Cap-Bon	Spring	28/04/2016	212224	170176	142142	150150	194202	127127	G164	1
8	pop1	potato	Cap-Bon	Spring	28/04/2016	212240	176178	142142	156156	194208	127127	G302	1
9	pop1	potato	Cap-Bon	Spring	28/04/2016	224240	174178	138148	162162	186194	153153	G328	3
10	pop1	potato	Cap-Bon	Spring	28/04/2016	238240	170172	136148	168168	186194	125125	G390	1
11	pop1	potato	Cap-Bon	Spring	30/04/2016	224240	178180	142148	150154	188188	129129	G331	5
12	pop1	potato	Cap-Bon	Spring	30/04/2016	224240	174178	138148	162162	186194	153153	G328	
13	pop1	potato	Cap-Bon	Spring	30/04/2016	212232	172176	136136	168168	188194	127149	G220	1
14	pop1	potato	Cap-Bon	Spring	30/04/2016	212212	178178	136142	152152	194204	127127	G125	1
15	pop1	potato	Cap-Bon	Spring	30/04/2016	212212	178178	138138	164164	190192	149153	G128	1
17	pop1	potato	Cap-Bon	Spring	05/05/2016	212240	178180	138138	150150	188194	151151	G305	1
18	pop1	potato	Cap-Bon	Spring	05/05/2016	212212	172176	142148	162164	192196	127149	G77	1
19	pop1	potato	Cap-Bon	Spring	05/05/2016	238240	170172	136142	168168	186194	129129	G389	1
20	pop1	potato	Cap-Bon	Spring	05/05/2016	212212	174176	136136	150150	194194	129147	G93	1
21	pop1	potato	Cap-Bon	Spring	05/05/2016	224240	178180	142148	150154	188188	129129	G331	
22	pop1	potato	Cap-Bon	Spring	05/05/2016	196212	170178	136148	162164	192194	149151	G6	1
23	pop1	potato	Cap-Bon	Spring	05/05/2016	224242	178180	142148	150154	188188	129129	G337	1

24	pop1	potato	Cap-Bon	Spring	05/05/2016	196212	172178	136148	168168	192196	149151	G13	1
25	pop1	potato	Cap-Bon	Spring	12/05/2016	224240	178180	142148	150154	188188	129129	G331	
26	pop1	potato	Cap-Bon	Spring	12/05/2016	238240	170172	136142	168168	186194	125125	G388	
27	pop1	potato	Cap-Bon	Spring	12/05/2016	224240	178180	142148	150154	188188	129129	G331	
28	pop1	potato	Cap-Bon	Spring	12/05/2016	224240	174178	138148	162162	186194	153153	G328	
29	pop1	potato	Cap-Bon	Spring	12/05/2016	238240	170172	136142	168168	186194	125125	G388	
30	pop1	potato	Cap-Bon	Spring	12/05/2016	212212	178178	136136	150150	194194	129129	G124	
31	pop1	potato	Cap-Bon	Spring	12/05/2016	238240	170172	136142	168168	186194	125125	G388	
32	pop1	potato	Cap-Bon	Spring	12/05/2016	238240	172172	136136	168168	186194	125125	G391	1
33	pop1	potato	Cap-Bon	Spring	12/05/2016	224240	178180	142148	150154	188188	129129	G331	
34	pop1	potato	Cap-Bon	Spring	12/05/2016	212238	172176	138148	156156	180194	125127	G266	1
35	pop1	potato	Cap-Bon	Spring	12/05/2016	232240	172178	138142	156156	192192	151153	G378	1
36	pop1	potato	Cap-Bon	Spring	12/05/2016	212212	172178	142142	160160	190194	151151	G90	1
37	pop1	potato	Cap-Bon	Spring	12/05/2016	232240	170178	136140	158158	194208	151151	G367	1
38	pop1	potato	Cap-Bon	Spring	12/05/2016	212232	170176	138142	168168	188194	149149	G214	1
39	pop1	potato	Cap-Bon	Spring	12/05/2016	212212	176176	136142	150150	194194	129147	G103	1
40	pop2	peach	Cap-Bon	Spring	05/05/2016	196212	170178	136148	162164	192194	149151	G6	29
41	pop2	peach	Cap-Bon	Spring	05/05/2016	196212	170178	136148	162164	192194	149151	G6	
42	pop2	peach	Cap-Bon	Spring	05/05/2016	196212	170178	136148	162164	192194	149151	G6	
43	pop2	peach	Cap-Bon	Spring	05/05/2016	196212	170178	136148	162164	192194	149151	G6	
44	pop2	peach	Cap-Bon	Spring	05/05/2016	196212	170178	136148	162164	192194	149151	G6	
45	pop2	peach	Cap-Bon	Spring	05/05/2016	196212	170178	136148	162164	192194	149151	G6	
46	pop2	peach	Cap-Bon	Spring	05/05/2016	212212	172176	142148	162164	192196	127149	G77	4
47	pop2	peach	Cap-Bon	Spring	05/05/2016	196212	170178	136148	162164	192194	149151	G6	
48	pop2	peach	Cap-Bon	Spring	05/05/2016	212212	172178	136148	152152	188194	151153	G81	1
49	pop2	peach	Cap-Bon	Spring	05/05/2016	196212	170178	136148	162164	192194	149151	G6	

50	pop2	peach	Cap-Bon	Spring	05/05/2016	196212	170178	136148	162164	192194	149151	G6	
51	pop2	peach	Cap-Bon	Spring	05/05/2016	196212	170178	136148	162164	192194	149151	G6	
52	pop2	peach	Cap-Bon	Spring	05/05/2016	196212	170178	136148	162164	192194	149151	G6	
53	pop2	peach	Cap-Bon	Spring	05/05/2016	212212	172176	142148	162164	192196	127149	G77	
54	pop2	peach	Cap-Bon	Spring	05/05/2016	212212	170178	136148	162164	192194	149151	G61	1
55	pop2	peach	Cap-Bon	Spring	05/05/2016	196212	170178	136148	162164	192194	149151	G6	
56	pop2	peach	Cap-Bon	Spring	05/05/2016	196212	170178	136148	162164	192194	149151	G6	
57	pop2	peach	Cap-Bon	Spring	05/05/2016	196212	170178	136148	162164	192194	149151	G6	
58	pop2	peach	Cap-Bon	Spring	05/05/2016	196212	170178	136148	162164	192194	149151	G6	
59	pop2	peach	Cap-Bon	Spring	05/05/2016	212212	172178	138148	176176	188194	151153	G88	1
60	pop2	peach	Cap-Bon	Spring	05/05/2016	212212	172178	138148	152152	188194	151153	G84	2
61	pop2	peach	Cap-Bon	Spring	05/05/2016	196212	170178	136148	162164	192194	149151	G6	
62	pop2	peach	Cap-Bon	Spring	05/05/2016	212212	172176	142148	162164	192198	127149	G78	1
63	pop2	peach	Cap-Bon	Spring	05/05/2016	196212	170178	136148	162164	192194	149151	G6	
65	pop2	peach	Cap-Bon	Spring	05/05/2016	212212	172178	138148	152152	188194	151153	G84	
66	pop2	peach	Cap-Bon	Spring	05/05/2016	208208	172176	142148	162164	192196	127149	G36	1
67	pop2	peach	Cap-Bon	Spring	05/05/2016	196212	170178	142142	162164	192194	149151	G8	1
68	pop2	peach	Cap-Bon	Spring	05/05/2016	196212	170178	148148	162164	192194	149151	G9	1
69	pop2	peach	Cap-Bon	Spring	05/05/2016	196212	170178	136148	162164	192194	149151	G6	
70	pop2	peach	Cap-Bon	Spring	05/05/2016	196212	170178	136148	162164	192194	149151	G6	
71	pop2	peach	Cap-Bon	Spring	05/05/2016	196212	170178	136148	162164	192194	149151	G6	
72	pop2	peach	Cap-Bon	Spring	05/05/2016	212212	172178	138148	152152	188194	151151	G83	1
73	pop2	peach	Cap-Bon	Spring	05/05/2016	196212	170178	136148	162164	192194	149151	G6	
74	pop2	peach	Cap-Bon	Spring	05/05/2016	212212	172178	138148	152152	188196	151151	G85	1
75	pop2	peach	Cap-Bon	Spring	05/05/2016	212212	176178	142148	162162	194206	125127	G121	1
76	pop2	peach	Cap-Bon	Spring	05/05/2016	196212	170178	136148	162164	192194	149151	G6	

77	pop2	peach	Cap-Bon	Spring	05/05/2016	196212	170178	136148	162164	192194	149151	G6	
78	pop2	peach	Cap-Bon	Spring	05/05/2016	192212	172178	138148	154154	188194	151151	G1	1
79	pop2	peach	Cap-Bon	Spring	05/05/2016	212212	172176	142148	162164	192196	127149	G77	
80	pop2	peach	Cap-Bon	Spring	05/05/2016	196212	170178	136148	162164	192194	149151	G6	
81	pop2	peach	Cap-Bon	Spring	05/05/2016	196212	170178	136148	162164	192194	149151	G6	
82	pop2	peach	Cap-Bon	Spring	05/05/2016	212212	172176	142148	162164	192196	127149	G77	
83	pop2	peach	Cap-Bon	Spring	05/05/2016	196212	170178	136148	162164	192194	149151	G6	
84	pop2	peach	Cap-Bon	Spring	05/05/2016	196212	170178	136148	162164	192194	149151	G6	
85	pop2	peach	Cap-Bon	Spring	05/05/2016	196212	172178	136148	160160	194196	149151	G12	1
86	pop2	peach	Cap-Bon	Spring	05/05/2016	196212	172178	136148	160160	192196	149151	G11	1
87	pop2	peach	Cap-Bon	Spring	05/05/2016	212212	176178	142148	162162	194204	125127	G120	1
88	pop2	peach	Cap-Bon	Spring	05/05/2016	196212	170178	136148	162164	192194	149151	G6	
89	pop2	peach	Cap-Bon	Spring	05/05/2016	196212	170178	136148	162164	192194	149151	G6	
90	pop2	peach	Cap-Bon	Spring	05/05/2016	210212	172178	138148	152152	188194	151151	G40	1
91	pop2	peach	Cap-Bon	Spring	05/05/2016	196212	170178	136148	162164	192196	149151	G7	1
214	pop2	peach	Cap-Bon	Spring	10/05/2016	212212	176178	138148	168168	188204	145153	G118	1
216	pop2	peach	Cap-Bon	Spring	10/05/2016	212224	170176	138148	156156	188188	145153	G161	2
217	pop2	peach	Cap-Bon	Spring	10/05/2016	212224	170176	138148	156156	188188	145153	G161	
218	pop2	peach	Cap-Bon	Spring	10/05/2016	212238	170172	138148	156156	188194	145149	G257	1
219	pop2	peach	Cap-Bon	Spring	10/05/2016	212238	170172	136138	156156	188194	145149	G254	1
220	pop2	peach	Cap-Bon	Spring	10/05/2016	214224	172176	138138	168168	190196	127127	G310	1
221	pop2	peach	Cap-Bon	Spring	10/05/2016	212224	170176	138148	156156	188194	145155	G163	2
222	pop2	peach	Cap-Bon	Spring	10/05/2016	212224	170176	138148	156156	188194	145155	G163	
223	pop2	peach	Cap-Bon	Spring	10/05/2016	212212	170178	140148	156156	188192	145153	G64	1
224	pop2	peach	Cap-Bon	Spring	10/05/2016	212224	170176	138148	156156	188194	145153	G162	3
225	pop2	peach	Cap-Bon	Spring	10/05/2016	212224	170176	138148	156156	188194	145153	G162	

226	pop2	peach	Cap-Bon	Spring	10/05/2016	212224	170176	138148	156156	188194	145153	G162	
227	pop2	peach	Cap-Bon	Spring	10/05/2016	232240	170178	138148	162162	194206	151151	G376	1
112	pop3	peach	Cap-Bon	Spring	10/05/2013	212238	172178	142150	150150	188194	145147	G276	1
113	pop3	peach	Cap-Bon	Spring	10/05/2013	212224	176176	142142	164164	188192	149149	G182	7
114	pop3	peach	Cap-Bon	Spring	10/05/2013	212236	176178	142148	162162	188188	153155	G247	22
115	pop3	peach	Cap-Bon	Spring	10/05/2013	212238	172176	142150	164164	188194	145147	G269	3
116	pop3	peach	Cap-Bon	Spring	10/05/2013	212224	176176	142142	154154	188192	149149	G181	1
117	pop3	peach	Cap-Bon	Spring	10/05/2013	212238	178180	142150	164164	188194	145149	G298	2
118	pop3	peach	Cap-Bon	Spring	10/05/2013	212224	170176	138138	152154	188188	127127	G155	1
119	pop3	peach	Cap-Bon	Spring	10/05/2013	212212	172174	138148	150150	190194	151151	G72	1
120	pop3	peach	Cap-Bon	Spring	10/05/2013	212226	170170	138138	160162	192194	151151	G192	4
121	pop3	peach	Cap-Bon	Spring	10/05/2013	212226	170170	138138	160162	192194	151151	G192	
122	pop3	peach	Cap-Bon	Spring	10/05/2013	212238	172176	142150	164164	188194	145149	G270	2
123	pop3	peach	Cap-Bon	Spring	10/05/2013	196238	170176	136138	168168	188188	149149	G28	2
124	pop3	peach	Cap-Bon	Spring	10/05/2013	212236	170170	136138	166166	188206	125151	G233	1
125	pop3	peach	Cap-Bon	Spring	10/05/2013	212236	176176	136138	164164	188194	127149	G243	1
126	pop3	peach	Cap-Bon	Spring	10/05/2013	232232	172178	138138	168168	194208	127127	G354	1
127	pop3	peach	Cap-Bon	Spring	10/05/2013	196238	170176	136138	168168	188188	149149	G28	
128	pop3	peach	Cap-Bon	Spring	10/05/2013	212236	176178	142148	162162	188188	153155	G247	
129	pop3	peach	Cap-Bon	Spring	10/05/2013	212224	170174	138138	150150	188188	151153	G154	1
130	pop3	peach	Cap-Bon	Spring	10/05/2013	212238	172176	142150	164164	188194	145149	G270	
131	pop3	peach	Cap-Bon	Spring	10/05/2013	224238	178180	142142	164164	188192	149149	G324	1
132	pop3	peach	Cap-Bon	Spring	10/05/2013	212224	176176	142142	164164	188192	149149	G182	
133	pop3	peach	Cap-Bon	Spring	10/05/2013	212224	176176	142142	164164	188192	149149	G182	
134	pop3	peach	Cap-Bon	Spring	10/05/2013	212236	176178	142148	162162	188188	153155	G247	
135	pop3	peach	Cap-Bon	Spring	10/05/2013	212232	172178	138138	168168	194210	127127	G226	1

136	pop3	peach	Cap-Bon	Spring	10/05/2013	212226	170170	138138	160162	192194	151151	G192	
137	pop3	peach	Cap-Bon	Spring	10/05/2013	212236	176178	142148	162162	188188	153155	G247	
138	pop3	peach	Cap-Bon	Spring	10/05/2013	212238	176176	142142	150150	188192	147149	G285	1
139	pop3	peach	Cap-Bon	Spring	10/05/2013	212238	172176	142150	164164	188194	145147	G269	
140	pop3	peach	Cap-Bon	Spring	10/05/2013	212212	178178	138138	164164	202210	151151	G130	1
141	pop3	peach	Cap-Bon	Spring	10/05/2013	212224	176176	142142	164164	188192	149149	G182	
142	pop3	peach	Cap-Bon	Spring	10/05/2013	212224	170170	138138	162164	188188	127127	G149	1
143	pop3	peach	Cap-Bon	Spring	10/05/2013	212238	172176	142150	164164	188194	145147	G269	
144	pop3	peach	Cap-Bon	Spring	10/05/2013	196212	176178	138138	160160	190198	147149	G15	1
145	pop3	peach	Cap-Bon	Spring	10/05/2013	212238	174176	142150	164164	188194	145149	G281	1
146	pop3	peach	Cap-Bon	Spring	10/05/2013	212224	176176	142142	164164	188192	149149	G182	
148	pop3	peach	Cap-Bon	Spring	10/05/2013	212226	170170	138138	160162	192194	151151	G192	
149	pop3	peach	Cap-Bon	Spring	10/05/2013	212238	176178	142150	164164	188194	145149	G290	1
150	pop3	peach	Cap-Bon	Spring	10/05/2013	212224	176176	142142	164164	188192	149149	G182	
151	pop3	peach	Cap-Bon	Spring	10/05/2013	212236	176178	142148	162162	188188	153155	G247	
152	pop3	peach	Cap-Bon	Spring	10/05/2013	196212	176178	138138	160160	190198	149149	G16	1
153	pop3	peach	Cap-Bon	Spring	10/05/2013	212238	176180	142150	164164	188194	145149	G293	1
154	pop3	peach	Cap-Bon	Spring	10/05/2013	212224	176176	142142	164164	188192	149149	G182	
155	pop3	peach	Cap-Bon	Spring	10/05/2013	212238	178180	142150	164164	188194	145149	G298	
156	pop3	peach	Cap-Bon	Spring	10/05/2013	212238	172176	142150	150150	188194	145149	G268	1
157	pop3	peach	Cap-Bon	Spring	30/05/2013	196238	170170	136138	168168	188188	151153	G24	1
158	pop3	peach	Cap-Bon	Spring	30/05/2013	212236	180180	142148	162162	188188	153155	G252	1
159	pop3	peach	Cap-Bon	Spring	30/05/2013	212236	176178	142148	162162	188194	153155	G248	2
160	pop3	peach	Cap-Bon	Spring	30/05/2013	212236	176178	142148	162162	188188	153155	G247	
161	pop3	peach	Cap-Bon	Spring	30/05/2013	212236	174174	142148	156156	180188	151153	G242	1
162	pop3	peach	Cap-Bon	Spring	30/05/2013	212236	176178	142148	162162	188188	153155	G247	

163	pop3	peach	Cap-Bon	Spring	30/05/2013	212236	176178	142148	162162	188188	153155	G247	
164	pop3	peach	Cap-Bon	Spring	30/05/2013	212212	174174	148148	160160	190190	151151	G92	1
165	pop3	peach	Cap-Bon	Spring	30/05/2013	212236	178180	142148	150154	194194	153153	G251	1
166	pop3	peach	Cap-Bon	Spring	30/05/2013	212236	176178	142148	162162	188188	153155	G247	
168	pop3	peach	Cap-Bon	Spring	30/05/2013	196238	170172	136138	164164	188208	149149	G26	1
169	pop3	peach	Cap-Bon	Spring	30/05/2013	212236	176178	142148	162162	188188	153155	G247	
170	pop3	peach	Cap-Bon	Spring	30/05/2013	212236	176178	142148	168168	186188	151153	G249	1
171	pop3	peach	Cap-Bon	Spring	30/05/2013	212236	172174	142148	162162	188190	153153	G239	1
172	pop3	peach	Cap-Bon	Spring	30/05/2013	212212	174176	138148	150150	190190	153153	G97	1
173	pop3	peach	Cap-Bon	Spring	30/05/2013	242242	176178	142148	160162	188188	159159	G397	1
174	pop3	peach	Cap-Bon	Spring	30/05/2013	196238	174176	146148	156156	186186	151153	G31	1
175	pop3	peach	Cap-Bon	Spring	30/05/2013	212232	176178	138138	150152	188188	153153	G229	1
176	pop3	peach	Cap-Bon	Spring	30/05/2013	212236	176178	142148	162162	188188	153155	G247	
177	pop3	peach	Cap-Bon	Spring	30/05/2013	212236	176178	142148	162162	188188	153155	G247	
178	pop3	peach	Cap-Bon	Spring	30/05/2013	212236	176178	142148	162162	188188	153155	G247	
181	pop3	peach	Cap-Bon	Spring	30/05/2013	212236	176178	142148	162162	188188	153155	G247	
182	pop3	peach	Cap-Bon	Spring	30/05/2013	212236	176178	142148	162162	188188	153155	G247	
183	pop3	peach	Cap-Bon	Spring	30/05/2013	212236	176178	142148	162162	188188	153155	G247	
184	pop3	peach	Cap-Bon	Spring	30/05/2013	212236	176178	142148	162162	188188	153155	G247	
185	pop3	peach	Cap-Bon	Spring	30/05/2013	212236	176178	142148	162162	188194	153155	G248	
186	pop3	peach	Cap-Bon	Spring	30/05/2013	212236	176178	142148	162162	188188	153155	G247	
187	pop3	peach	Cap-Bon	Spring	30/05/2013	212236	176178	142148	162162	188188	153155	G247	
188	pop3	peach	Cap-Bon	Spring	30/05/2013	208234	176178	148148	162162	188188	153155	G38	1
189	pop3	peach	Cap-Bon	Spring	30/05/2013	212236	176178	142148	162162	188188	153155	G247	
190	pop3	peach	Cap-Bon	Spring	30/05/2013	212236	176178	142148	162162	188188	153155	G247	
191	pop3	peach	Cap-Bon	Spring	30/05/2013	212236	176178	142148	162162	188188	153155	G247	

192	pop3	peach	Cap-Bon	Spring	30/05/2013	212236	176178	142148	162162	188188	152154	G246	1
528	pop4	potato	Cap-Bon	autumn	30/11/2012	224240	172172	136142	154156	186200	127145	G326	1
529	pop4	potato	Cap-Bon	autumn	30/11/2012	212232	170176	138142	154156	188196	151151	G211	1
530	pop4	potato	Cap-Bon	autumn	30/11/2012	232236	170172	148148	154156	192194	149151	G359	1
531	pop4	potato	Cap-Bon	autumn	30/11/2012	212212	170178	136148	152154	188190	127127	G60	1
532	pop4	potato	Cap-Bon	autumn	30/11/2012	226238	176178	142142	162164	196196	159159	G343	1
533	pop4	potato	Cap-Bon	autumn	30/11/2012	232240	170172	138142	154156	194206	151151	G365	1
534	pop4	potato	Cap-Bon	autumn	30/11/2012	212240	178178	138138	150152	188194	149151	G304	1
535	pop4	potato	Cap-Bon	autumn	30/11/2012	224238	178178	138142	152152	194194	153155	G323	1
536	pop4	potato	Cap-Bon	autumn	30/11/2012	226240	170172	138138	150150	194194	153155	G344	1
537	pop4	potato	Cap-Bon	autumn	30/11/2012	232240	170178	138142	150152	194208	153153	G372	1
538	pop4	potato	Cap-Bon	autumn	30/11/2012	212232	170176	138142	154156	188194	127149	G210	1
539	pop4	potato	Cap-Bon	autumn	30/11/2012	212232	172172	138142	150150	192194	151153	G219	1
540	pop4	potato	Cap-Bon	autumn	30/11/2012	212226	172178	136138	152154	194206	147147	G196	1
541	pop4	potato	Cap-Bon	autumn	30/11/2012	212226	170178	136138	152152	196206	145145	G194	1
542	pop4	potato	Cap-Bon	autumn	30/11/2012	224240	178180	142148	154156	188188	127149	G336	2
543	pop4	potato	Cap-Bon	autumn	30/11/2012	224240	178180	142148	154156	188188	127149	G336	
544	pop4	potato	Cap-Bon	autumn	30/11/2012	212218	170178	136138	150150	188194	153155	G145	1
545	pop4	potato	Cap-Bon	autumn	30/11/2012	226238	170172	138138	152152	194194	153155	G342	1
546	pop4	potato	Cap-Bon	autumn	30/11/2012	224240	178180	142148	150154	188188	129129	G331	1
547	pop4	potato	Cap-Bon	autumn	30/11/2012	212232	170176	138142	150154	188194	127127	G209	1
548	pop4	potato	Cap-Bon	autumn	30/11/2012	232240	170178	138142	150150	194206	151153	G368	1
549	pop4	potato	Cap-Bon	autumn	30/11/2012	212232	170172	138142	152152	194194	153155	G206	1
550	pop4	potato	Cap-Bon	autumn	30/11/2012	232240	170172	138142	150150	194208	153155	G364	1
551	pop4	potato	Cap-Bon	autumn	30/11/2012	232236	170172	138140	150150	192194	153155	G357	1
590	pop5	peach	Cap-Bon	autumn	30/11/2012	196196	178180	138148	164164	194194	127127	G3	1

591	pop5	peach	Cap-Bon	autumn	30/11/2012	196212	176176	138138	162162	188194	145145	G14	1
592	pop5	peach	Cap-Bon	autumn	30/11/2012	212212	172172	138148	150150	188206	151153	G68	1
593	pop5	peach	Cap-Bon	autumn	30/11/2012	212236	176178	138138	156156	190194	149151	G245	1
594	pop5	peach	Cap-Bon	autumn	30/11/2012	196196	178180	138148	164164	194204	127127	G4	1
595	pop5	peach	Cap-Bon	autumn	30/11/2012	212212	172176	142142	152152	194196	127127	G76	1
596	pop5	peach	Cap-Bon	autumn	30/11/2012	212212	174176	138142	164164	194202	153155	G96	1
597	pop5	peach	Cap-Bon	autumn	30/11/2012	212224	178180	138148	152152	190194	127127	G191	1
598	pop5	peach	Cap-Bon	autumn	30/11/2012	212236	170178	138148	164164	192196	145149	G238	1
599	pop5	peach	Cap-Bon	autumn	30/11/2012	224224	178178	136138	156156	188194	145149	G315	1
344	pop6	potato	Cap-Bon	Winter	08/12/2011	212232	170176	138142	150150	188194	127127	G208	1
345	pop6	potato	Cap-Bon	Winter	08/12/2011	212240	176178	138138	150150	188194	151151	G301	1
346	pop6	potato	Cap-Bon	Winter	08/12/2011	232240	184186	138142	154156	194208	151153	G384	1
347	pop6	potato	Cap-Bon	Winter	08/12/2011	212232	170176	138142	168168	188194	149149	G214	4
348	pop6	potato	Cap-Bon	Winter	08/12/2011	212232	172178	138138	210212	192194	145149	G227	1
349	pop6	potato	Cap-Bon	Winter	08/12/2011	212212	178178	142142	152152	196204	127127	G135	1
350	pop6	potato	Cap-Bon	Winter	08/12/2011	212238	176178	138138	150150	180194	125127	G287	1
351	pop6	potato	Cap-Bon	Winter	08/12/2011	212238	172178	136138	206208	188194	127127	G271	1
352	pop6	potato	Cap-Bon	Winter	08/12/2011	212232	172176	138142	166168	188194	149149	G222	1
353	pop6	potato	Cap-Bon	Winter	08/12/2011	232240	184186	138142	154156	194208	151151	G383	2
354	pop6	potato	Cap-Bon	Winter	08/12/2011	212230	170170	148148	168168	188194	159159	G200	1
355	pop6	potato	Cap-Bon	Winter	08/12/2011	232240	184186	138142	154156	194208	151151	G383	
356	pop6	potato	Cap-Bon	Winter	08/12/2011	232236	174176	138140	150150	192194	151153	G361	1
357	pop6	potato	Cap-Bon	Winter	08/12/2011	212230	172172	138138	159159	188194	149149	G201	1
358	pop6	potato	Cap-Bon	Winter	08/12/2011	212232	170176	138142	168168	188194	149149	G214	
360	pop6	potato	Cap-Bon	Winter	08/12/2011	212232	170176	138142	168168	188194	149149	G214	
361	pop6	potato	Cap-Bon	Winter	08/12/2011	212232	172176	138142	168168	188194	149149	G223	1

362	pop6	potato	Cap-Bon	Winter	08/12/2011	224240	178180	142142	150150	188188	129129	G329	1
363	pop6	potato	Cap-Bon	Winter	08/12/2011	212232	170176	138142	168168	188194	149149	G214	
364	pop6	potato	Cap-Bon	Winter	08/12/2011	238242	176178	138138	162162	190194	155155	G394	1
365	pop6	potato	Cap-Bon	Winter	08/12/2011	226240	176178	138138	176178	194194	153155	G350	1
366	pop6	potato	Cap-Bon	Winter	08/12/2011	212232	170176	138142	166168	188194	149149	G212	1
367	pop6	potato	Cap-Bon	Winter	08/12/2011	238242	176178	138138	156156	190194	155155	G393	1
324	pop7	peach	Cap-Bon	Winter	08/12/2011	236238	178178	138138	154154	190194	149151	G386	1
325	pop7	peach	Cap-Bon	Winter	08/12/2011	212212	176176	136142	152152	194194	151151	G104	1
327	pop7	peach	Cap-Bon	Winter	08/12/2011	212238	176178	138144	162162	194200	151153	G288	1
328	pop7	peach	Cap-Bon	Winter	08/12/2011	212212	172174	138142	152152	192194	153155	G71	1
330	pop7	peach	Cap-Bon	Winter	08/12/2011	212212	174176	136142	150150	194204	149151	G94	1
331	pop7	peach	Cap-Bon	Winter	08/12/2011	212232	176178	138138	150152	188194	149149	G230	1
333	pop7	peach	Cap-Bon	Winter	08/12/2011	196196	176178	136138	152152	190194	127127	G2	1
335	pop7	peach	Cap-Bon	Winter	08/12/2011	224236	170178	138142	150150	206206	151151	G320	1
336	pop7	peach	Cap-Bon	Winter	08/12/2011	212212	170172	136138	152152	194194	151151	G46	1
337	pop7	peach	Cap-Bon	Winter	08/12/2011	212232	172176	138142	168168	188196	149149	G224	1
340	pop7	peach	Cap-Bon	Winter	01/12/2011	196238	176178	138142	208210	188194	149151	G32	1
341	pop7	peach	Cap-Bon	Winter	01/12/2011	212212	178178	138138	164164	194194	149149	G129	1
343	pop7	peach	Cap-Bon	Winter	01/12/2011	212212	178178	138142	208210	188204	149149	G133	1
513	pop7	peach	Cap-Bon	Winter	22/12/2011	212224	172178	138142	150150	194196	153155	G175	1
514	pop7	peach	Cap-Bon	Winter	22/12/2011	212236	170170	138142	162162	194206	127153	G234	1
515	pop7	peach	Cap-Bon	Winter	22/12/2011	212232	170170	142142	168168	194194	151153	G203	1
516	pop7	peach	Cap-Bon	Winter	22/12/2011	212212	172174	138138	168168	188194	149151	G70	1
517	pop7	peach	Cap-Bon	Winter	22/12/2011	212212	176178	138138	166166	188188	151153	G113	1
518	pop7	peach	Cap-Bon	Winter	22/12/2011	212212	176176	136138	150150	188194	155155	G101	1
519	pop7	peach	Cap-Bon	Winter	22/12/2011	212238	176176	138148	150154	194194	125125	G284	1

520	pop7	peach	Cap-Bon	Winter	22/12/2011	196224	176178	140140	152154	194194	145149	G20	1
521	pop7	peach	Cap-Bon	Winter	22/12/2011	232236	170172	138140	152152	192194	153155	G358	1
522	pop7	peach	Cap-Bon	Winter	22/12/2011	212212	172178	144148	152152	188204	127127	G91	1
523	pop7	peach	Cap-Bon	Winter	22/12/2011	196224	176178	138142	152154	194194	145141	G19	1
524	pop7	peach	Cap-Bon	Winter	22/12/2011	212212	170176	138142	152152	188194	145145	G57	1
525	pop7	peach	Cap-Bon	Winter	22/12/2011	212238	170176	138142	154160	188192	145145	G260	1
526	pop7	peach	Cap-Bon	Winter	22/12/2011	212236	172176	148148	154156	194198	149151	G241	1
527	pop7	peach	Cap-Bon	Winter	22/12/2011	212232	172176	142142	150150	188194	127127	G225	1
577	pop8	potato	Jendouba	Spring	10/05/2016	212224	170180	138142	150150	194204	151153	G167	1
578	pop8	potato	Jendouba	Spring	10/05/2016	212224	176178	142142	150150	188194	155155	G186	2
579	pop8	potato	Jendouba	Spring	10/05/2016	212212	176178	138142	162162	194194	127145	G116	1
580	pop8	potato	Jendouba	Spring	10/05/2016	232240	170178	138142	150150	194208	151151	G369	1
581	pop8	potato	Jendouba	Spring	10/05/2016	212212	172178	138148	154154	194194	149151	G86	1
582	pop8	potato	Jendouba	Spring	10/05/2016	232240	170178	138142	150150	194208	151153	G370	1
583	pop8	potato	Jendouba	Spring	10/05/2016	224240	178180	142148	152154	188188	129149	G333	1
584	pop8	potato	Jendouba	Spring	10/05/2016	212224	176178	142142	150150	188194	155155	G186	
97	pop9	peach	Jendouba	Spring	10/05/2016	212224	172176	138148	154154	188198	149151	G173	1
98	pop9	peach	Jendouba	Spring	10/05/2016	212224	170176	138148	154154	188194	145155	G159	3
99	pop9	peach	Jendouba	Spring	10/05/2016	212224	170176	138148	154154	188194	145155	G159	
100	pop9	peach	Jendouba	Spring	10/05/2016	212238	170172	136138	156156	188194	154149	G255	1
101	pop9	peach	Jendouba	Spring	10/05/2016	212238	172178	142142	172176	188206	145145	G275	1
102	pop9	peach	Jendouba	Spring	10/05/2016	212238	172180	142142	172176	188206	145145	G278	2
103	pop9	peach	Jendouba	Spring	10/05/2016	212224	170176	138148	154154	188194	145155	G159	
104	pop9	peach	Jendouba	Spring	10/05/2016	214224	172176	138138	168168	190196	127151	G311	1
105	pop9	peach	Jendouba	Spring	10/05/2016	212238	170172	136138	150150	188194	145147	G253	2
106	pop9	peach	Jendouba	Spring	10/05/2016	212238	172180	142142	150150	188206	145145	G277	1

107	pop9	peach	Jendouba	Spring	10/05/2016	212224	172176	138148	154154	188196	145155	G172	1
108	pop9	peach	Jendouba	Spring	10/05/2016	212238	172174	136138	156158	188196	145149	G265	1
109	pop9	peach	Jendouba	Spring	10/05/2016	212238	170172	136138	150150	188194	145147	G253	
110	pop9	peach	Jendouba	Spring	10/05/2016	212238	172180	142142	172176	188206	145145	G278	
111	pop9	peach	Jendouba	Spring	10/05/2016	212224	170176	138148	154154	188196	149151	G160	1
228	pop10	potato	Jendouba	Winter	03/01/2013	224240	178180	142148	150154	188188	129129	G331	4
229	pop10	potato	Jendouba	Winter	03/01/2013	212224	176178	142142	156156	188194	149149	G187	1
232	pop10	potato	Jendouba	Winter	03/01/2013	212212	172178	138148	168168	194206	127127	G87	1
233	pop10	potato	Jendouba	Winter	03/01/2013	224240	178180	142148	150154	188188	129129	G331	
234	pop10	potato	Jendouba	Winter	03/01/2013	212212	170172	138142	156156	188188	153153	G50	1
235	pop10	potato	Jendouba	Winter	03/01/2013	224240	178180	142148	150154	188192	129129	G332	1
236	pop10	potato	Jendouba	Winter	03/01/2013	226242	176176	132138	156156	186186	153155	G351	1
237	pop10	potato	Jendouba	Winter	03/01/2013	232240	1781780	138142	162162	188194	153155	G380	1
238	pop10	potato	Jendouba	Winter	03/01/2013	232240	170178	138142	150150	194206	151153	G368	1
240	pop10	potato	Jendouba	Winter	03/01/2013	212224	176178	130138	150150	194202	151153	G183	1
241	pop10	potato	Jendouba	Winter	03/01/2013	232232	172172	138142	164164	194194	153153	G353	1
242	pop10	potato	Jendouba	Winter	03/01/2013	224240	178180	142148	154154	188188	149151	G335	1
243	pop10	potato	Jendouba	Winter	03/01/2013	224244	170178	138142	150150	194206	151153	G338	1
244	pop10	potato	Jendouba	Winter	03/01/2013	224240	178180	142148	150154	188188	129129	G331	
245	pop10	potato	Jendouba	Winter	03/01/2013	224240	178180	142148	150154	188188	129129	G331	
246	pop10	potato	Jendouba	Winter	03/01/2013	212224	170180	138142	154154	194202	149151	G169	1
247	pop10	potato	Jendouba	Winter	03/01/2013	212224	170180	138142	154154	188192	149151	G168	1
249	pop11	potato	Jendouba	Winter	20/12/2012	214226	172178	140142	162164	188188	151151	G312	1
252	pop11	potato	Jendouba	Winter	20/12/2012	212224	172176	138142	154156	190192	149151	G171	1
257	pop11	potato	Jendouba	Winter	20/12/2012	214234	172174	138142	176178	186188	159159	G313	1
600	pop11	potato	Jendouba	Winter	20/12/2012	232240	184186	138142	150150	196208	151151	G382	1

601	pop11	potato	Jendouba	Winter	20/12/2012	212224	178178	138142	150150	196204	151153	G189	1
602	pop11	potato	Jendouba	Winter	20/12/2012	224240	178180	142148	150152	188188	129129	G330	1
603	pop11	potato	Jendouba	Winter	20/12/2012	224240	178180	142148	154154	188188	129149	G334	8
604	pop11	potato	Jendouba	Winter	20/12/2012	224240	178180	142148	154154	188188	129149	G334	
605	pop11	potato	Jendouba	Winter	20/12/2012	224240	178180	142148	154154	188188	129149	G334	
606	pop11	potato	Jendouba	Winter	20/12/2012	240240	180180	138142	150150	196196	159159	G396	1
607	pop11	potato	Jendouba	Winter	20/12/2012	232240	184186	138142	154156	194208	151151	G383	2
608	pop11	potato	Jendouba	Winter	20/12/2012	224240	178180	142148	154154	188188	129149	G334	
609	pop11	potato	Jendouba	Winter	20/12/2012	232236	174174	138142	166168	192194	151153	G360	1
610	pop11	potato	Jendouba	Winter	20/12/2012	224240	178180	142148	154154	188188	129149	G334	
611	pop11	potato	Jendouba	Winter	20/12/2012	224240	178180	142148	154154	188188	129149	G334	
612	pop11	potato	Jendouba	Winter	20/12/2012	224240	178180	142148	154154	188188	129149	G334	
613	pop11	potato	Jendouba	Winter	20/12/2012	232240	170186	138142	160160	194208	151151	G377	1
614	pop11	potato	Jendouba	Winter	20/12/2012	224240	178180	142148	154154	188188	129149	G334	
615	pop11	potato	Jendouba	Winter	20/12/2012	212226	170176	136138	168168	194206	151151	G193	1
616	pop11	potato	Jendouba	Winter	20/12/2012	212212	178180	136138	160164	186190	153155	G139	1
617	pop11	potato	Jendouba	Winter	20/12/2012	196238	170176	138148	150152	188194	153153	G29	1
618	pop11	potato	Jendouba	Winter	20/12/2012	232240	184186	138142	154156	194208	151151	G383	
289	pop12	peach	Jendouba	Winter	20/12/2012	212212	176178	138142	156156	188196	153153	G114	1
290	pop12	peach	Jendouba	Winter	20/12/2012	210224	172178	144146	168168	192192	145149	G43	1
292	pop12	peach	Jendouba	Winter	20/12/2012	212212	176178	138138	162162	196198	149149	G112	1
294	pop12	peach	Jendouba	Winter	20/12/2012	212234	176178	148148	150152	190192	149149	G232	1
298	pop12	peach	Jendouba	Winter	20/12/2012	224224	172178	138138	152152	192200	147149	G314	1
299	pop12	peach	Jendouba	Winter	20/12/2012	212212	170180	136138	162162	194194	149151	G66	1
300	pop12	peach	Jendouba	Winter	20/12/2012	212242	178178	138138	162162	196196	151151	G308	1
302	pop12	peach	Jendouba	Winter	20/12/2012	212232	170178	138138	152152	194210	151151	G217	1

303	pop12	peach	Jendouba	Winter	20/12/2012	212212	178180	138138	178178	188204	149149	G140	1
304	pop12	peach	Jendouba	Winter	20/12/2012	212212	176178	138142	162162	194194	149149	G117	1
305	pop12	peach	Jendouba	Winter	20/12/2012	212232	174176	142142	172176	188192	149151	G228	1
306	pop12	peach	Jendouba	Winter	20/12/2012	212224	170176	138142	168168	188210	153153	G158	1
307	pop12	peach	Jendouba	Winter	20/12/2012	212236	178180	138138	156156	194196	149149	G250	1
309	pop12	peach	Jendouba	Winter	20/12/2012	212212	178178	138138	164164	188196	151151	G127	1
310	pop12	peach	Jendouba	Winter	20/12/2012	212212	176178	138142	162162	188194	149149	G115	1
311	pop12	peach	Jendouba	Winter	20/12/2012	212212	176178	142142	166166	190194	149149	G119	1
312	pop12	peach	Jendouba	Winter	20/12/2012	212238	172176	142142	164164	190196	149151	G267	1
313	pop12	peach	Jendouba	Winter	20/12/2012	212238	176176	136136	156156	188206	149149	G282	1
315	pop12	peach	Jendouba	Winter	20/12/2012	212244	178180	142142	156156	188196	149149	G309	1
316	pop12	peach	Jendouba	Winter	20/12/2012	212212	170178	138142	152156	188206	127127	G63	1
318	pop12	peach	Jendouba	Winter	20/12/2012	224224	178180	138138	152152	188192	149151	G316	1
319	pop12	peach	Jendouba	Winter	20/12/2012	224238	176178	138138	156156	194198	149149	G322	1
320	pop12	peach	Jendouba	Winter	20/12/2012	232242	170172	136136	156156	194194	149149	G385	1
321	pop12	peach	Jendouba	Winter	20/12/2012	212212	170172	142142	168168	192194	149151	G52	1
322	pop12	peach	Jendouba	Winter	20/12/2012	212212	180180	138142	156156	192194	127127	G141	1
323	pop12	peach	Jendouba	Winter	20/12/2012	196232	172174	136138	174176	194208	151151	G23	1
438	pop13	potato	Jendouba	Winter	13/12/2011	212236	170176	142148	164166	194196	145149	G236	1
439	pop13	potato	Jendouba	Winter	13/12/2011	212212	178178	138138	150150	188194	151153	G126	1
440	pop13	potato	Jendouba	Winter	13/12/2011	212224	178180	136136	154156	194194	127127	G190	1
441	pop13	potato	Jendouba	Winter	13/12/2011	196238	170178	136148	162164	194194	155155	G30	1
442	pop13	potato	Jendouba	Winter	13/12/2011	212224	174176	138138	166166	194194	155155	G179	1
443	pop13	potato	Jendouba	Winter	13/12/2011	212224	172178	138150	164164	188188	153155	G177	1
444	pop13	potato	Jendouba	Winter	13/12/2011	238240	176178	138142	152152	194194	153155	G392	1
445	pop13	potato	Jendouba	Winter	13/12/2011	232240	170178	138142	150150	196208	151153	G371	3

446	pop13	potato	Jendouba	Winter	13/12/2011	232240	170178	138142	150150	194208	151153	G370	14
447	pop13	potato	Jendouba	Winter	13/12/2011	224244	170178	148148	150150	194208	151153	G340	2
448	pop13	potato	Jendouba	Winter	13/12/2011	232240	170178	138142	150150	194208	151153	G370	
449	pop13	potato	Jendouba	Winter	13/12/2011	232240	184186	138142	154156	194208	151151	G383	1
450	pop13	potato	Jendouba	Winter	13/12/2011	212224	170176	138142	152154	188188	127127	G157	1
451	pop13	potato	Jendouba	Winter	13/12/2011	232240	170178	138142	150150	194208	151153	G370	
452	pop13	potato	Jendouba	Winter	13/12/2011	224240	178180	142148	150154	188188	129129	G331	7
453	pop13	potato	Jendouba	Winter	13/12/2011	224226	170176	138148	152152	194194	153155	G318	1
454	pop13	potato	Jendouba	Winter	13/12/2011	224240	178180	142148	150152	188188	129129	G330	1
455	pop13	potato	Jendouba	Winter	13/12/2011	232240	170178	138142	150150	194208	151153	G370	
456	pop13	potato	Jendouba	Winter	13/12/2011	224244	170178	148148	152152	194208	153155	G341	1
457	pop13	potato	Jendouba	Winter	13/12/2011	232240	170178	138142	150150	196208	151153	G371	
458	pop13	potato	Jendouba	Winter	13/12/2011	232240	170178	138142	150150	196208	151153	G371	
459	pop13	potato	Jendouba	Winter	13/12/2011	224240	178180	142148	150154	188188	129129	G331	
460	pop13	potato	Jendouba	Winter	13/12/2011	224240	178180	142148	150154	188188	129129	G331	
461	pop13	potato	Jendouba	Winter	13/12/2011	224226	170176	138148	156156	194194	127127	G319	1
462	pop13	potato	Jendouba	Winter	13/12/2011	232240	170178	138142	152152	194208	151153	G374	1
463	pop13	potato	Jendouba	Winter	13/12/2011	224240	178180	142148	150154	188188	129129	G331	
464	pop13	potato	Jendouba	Winter	13/12/2011	232240	170178	138142	150150	194208	151153	G370	
465	pop13	potato	Jendouba	Winter	13/12/2011	232240	170178	138142	150150	194208	151153	G370	
466	pop13	potato	Jendouba	Winter	13/12/2011	224240	178180	142148	150154	188188	129129	G331	
467	pop13	potato	Jendouba	Winter	13/12/2011	224240	178180	142148	150154	188188	129129	G331	
468	pop13	potato	Jendouba	Winter	13/12/2011	226240	170172	138138	150152	194194	153155	G345	1
469	pop13	potato	Jendouba	Winter	13/12/2011	212226	172180	138142	154156	194204	149151	G197	1
470	pop13	potato	Jendouba	Winter	13/12/2011	232240	170178	138142	150150	194208	151153	G370	
471	pop13	potato	Jendouba	Winter	13/12/2011	232240	170178	138142	150150	194208	151153	G370	

472	pop13	potato	Jendouba	Winter	13/12/2011	232240	170178	138142	150150	194208	151153	G370	
473	pop13	potato	Jendouba	Winter	13/12/2011	226240	170172	138138	152152	194194	149153	G346	1
474	pop13	potato	Jendouba	Winter	13/12/2011	232240	170178	138142	150150	194208	151153	G370	
475	pop13	potato	Jendouba	Winter	13/12/2011	226240	170172	138138	152152	194194	153155	G347	1
476	pop13	potato	Jendouba	Winter	13/12/2011	232240	170178	138142	150150	194208	151153	G370	
477	pop13	potato	Jendouba	Winter	13/12/2011	232240	170178	138142	150150	194208	151153	G370	
478	pop13	potato	Jendouba	Winter	13/12/2011	224244	170178	148148	150150	194208	151153	G340	
479	pop13	potato	Jendouba	Winter	13/12/2011	232240	170178	138142	150150	194208	151153	G370	
480	pop13	potato	Jendouba	Winter	13/12/2011	224240	178180	142148	150154	188188	129129	G331	
573	pop13	potato	Jendouba	Winter	13/12/2011	232240	170178	138142	150150	194208	151153	G370	
574	pop13	potato	Jendouba	Winter	13/12/2011	226240	170172	138138	154156	194194	149151	G349	1
575	pop13	potato	Jendouba	Winter	13/12/2011	224244	170178	148148	150150	194208	151151	G339	1
576	pop13	potato	Jendouba	Winter	14/12/2011	226240	170172	138138	152152	196196	153155	G348	1
385	pop14	peach	Jendouba	Winter	13/12/2011	212232	170176	138148	152154	194200	145149	G215	1
386	pop14	peach	Jendouba	Winter	13/12/2011	212212	176176	138148	168168	188196	153155	G107	1
387	pop14	peach	Jendouba	Winter	13/12/2011	196212	172176	136140	168168	194196	151153	G10	1
388	pop14	peach	Jendouba	Winter	13/12/2011	238238	170178	138148	154156	200200	149151	G387	1
389	pop14	peach	Jendouba	Winter	13/12/2011	196242	170170	138138	164164	192194	153155	G33	1
390	pop14	peach	Jendouba	Winter	13/12/2011	224238	172178	142142	154156	188192	125127	G321	1
391	pop14	peach	Jendouba	Winter	13/12/2011	212238	170176	138148	162162	188194	153155	G263	1
392	pop14	peach	Jendouba	Winter	13/12/2011	196212	176178	138142	152152	194206	127127	G17	1
393	pop14	peach	Jendouba	Winter	13/12/2011	212224	178178	136136	154154	194194	127127	G188	1
394	pop14	peach	Jendouba	Winter	13/12/2011	212238	170176	138142	162162	188194	153155	G261	1
395	pop14	peach	Jendouba	Winter	13/12/2011	212238	176178	148148	150152	194194	127127	G291	1
396	pop14	peach	Jendouba	Winter	13/12/2011	212212	172176	138138	168168	194204	145153	G74	1
397	pop14	peach	Jendouba	Winter	13/12/2011	212212	170176	138142	162162	188194	153153	G58	1

398	pop14	peach	Jendouba	Winter	13/12/2011	212238	176178	136138	164164	192194	155155	G286	1
399	pop14	peach	Jendouba	Winter	13/12/2011	212212	178178	138142	162162	188194	153155	G132	1
400	pop14	peach	Jendouba	Winter	13/12/2011	212212	178180	136138	152154	188204	127127	G138	1
401	pop14	peach	Jendouba	Winter	13/12/2011	212240	172178	142148	164164	188206	151151	G300	1
402	pop14	peach	Jendouba	Winter	13/12/2011	232238	170170	138138	150150	194196	151153	G362	1
403	pop14	peach	Jendouba	Winter	13/12/2011	212212	174178	142148	152152	190194	153155	G100	1
404	pop14	peach	Jendouba	Winter	13/12/2011	212212	172174	142144	152152	194198	153155	G73	1
405	pop14	peach	Jendouba	Winter	13/12/2011	212212	176178	136138	152152	192198	153155	G110	1
407	pop14	peach	Jendouba	Winter	13/12/2011	196212	176178	138148	152152	188194	153155	G18	1
408	pop14	peach	Jendouba	Winter	13/12/2011	212212	172172	148148	150150	188198	151153	G69	1
409	pop14	peach	Jendouba	Winter	13/12/2011	212238	176178	138148	154154	188194	127127	G289	1
410	pop14	peach	Jendouba	Winter	13/12/2011	212212	178178	138148	154156	188194	127127	G134	1
411	pop14	peach	Jendouba	Winter	13/12/2011	212236	176176	142148	164164	194196	145149	G244	1
412	pop14	peach	Jendouba	Winter	13/12/2011	212224	170178	136142	156156	188194	151153	G165	1
413	pop14	peach	Jendouba	Winter	13/12/2011	238244	172178	142148	164164	188196	153155	G395	1
414	pop14	peach	Jendouba	Winter	13/12/2011	212212	174176	138138	154156	188194	127127	G95	1
415	pop14	peach	Jendouba	Winter	13/12/2011	212212	176178	142148	166168	194194	151153	G122	1
416	pop14	peach	Jendouba	Winter	13/12/2011	212226	178178	142142	152152	188190	153153	G198	1
417	pop14	peach	Jendouba	Winter	13/12/2011	212212	172178	136136	152152	194194	151153	G80	1
418	pop14	peach	Jendouba	Winter	13/12/2011	212212	176176	136138	156156	192206	153153	G102	1
419	pop14	peach	Jendouba	Winter	13/12/2011	212212	176176	142142	154156	190200	149151	G109	1
420	pop14	peach	Jendouba	Winter	13/12/2011	212218	176178	142142	162164	190194	153155	G146	1
421	pop14	peach	Jendouba	Winter	13/12/2011	212212	178178	138142	152154	192194	145149	G131	1
422	pop14	peach	Jendouba	Winter	13/12/2011	196226	170178	138138	150150	188188	151153	G21	1
424	pop14	peach	Jendouba	Winter	13/12/2011	212212	178178	142142	168168	188198	151153	G136	1
425	pop14	peach	Jendouba	Winter	13/12/2011	212238	170176	136142	164164	188194	153155	G258	1

426	pop14	peach	Jendouba	Winter	13/12/2011	212240	172174	138138	152152	188194	153155	G299	1
427	pop14	peach	Jendouba	Winter	13/12/2011	212238	178178	138142	150150	194194	151153	G294	1
428	pop14	peach	Jendouba	Winter	13/12/2011	212212	176178	136142	150150	194198	151153	G111	1
429	pop14	peach	Jendouba	Winter	13/12/2011	196212	170172	138142	156156	188196	127131	G5	1
430	pop14	peach	Jendouba	Winter	13/12/2011	212212	176178	148150	150150	188202	153155	G123	1
431	pop14	peach	Jendouba	Winter	13/12/2011	212224	176176	138144	150150	194198	153155	G180	1
432	pop14	peach	Jendouba	Winter	13/12/2011	232238	176178	136148	152154	188194	127145	G363	1
433	pop14	peach	Jendouba	Winter	13/12/2011	212212	176176	142142	150150	190198	151153	G108	1
434	pop14	peach	Jendouba	Winter	13/12/2011	212224	172178	138150	164164	188188	153153	G176	1
435	pop14	peach	Jendouba	Winter	13/12/2011	212212	172178	136136	150150	194194	151153	G79	1
436	pop14	peach	Jendouba	Winter	13/12/2011	212226	178180	138142	162162	188204	145145	G199	1
437	pop14	peach	Jendouba	Winter	13/12/2011	212212	172174	138142	152152	192194	153155	G71	1
585	pop15	potato	Kairouan	Spring	27/04/2016	232232	178178	136138	152154	194194	129129	G355	1
586	pop15	potato	Kairouan	Spring	27/04/2016	212238	170176	138138	164164	188210	149151	G259	1
587	pop15	potato	Kairouan	Spring	27/04/2016	212238	172178	138138	152152	194194	127127	G272	1
588	pop15	potato	Kairouan	Spring	27/04/2016	212224	172180	138148	154154	194204	149151	G178	1
589	pop15	potato	Kairouan	Spring	27/04/2016	224240	174178	138148	162162	186194	153153	G328	1
193	pop16	peach	Kairouan	Spring	27/04/2016	212238	176176	138138	168168	188194	145149	G283	1
195	pop16	peach	Kairouan	Spring	27/04/2016	212232	170176	148148	150150	188188	127147	G216	2
196	pop16	peach	Kairouan	Spring	27/04/2016	212232	168172	148148	154154	188190	125151	G202	1
197	pop16	peach	Kairouan	Spring	27/04/2016	212212	172178	138138	162162	194196	125125	G82	1
198	pop16	peach	Kairouan	Spring	27/04/2016	212238	174176	136142	168168	188188	145145	G279	1
199	pop16	peach	Kairouan	Spring	27/04/2016	212232	170176	148148	150150	188188	127147	G216	
200	pop16	peach	Kairouan	Spring	27/04/2016	212214	172178	138138	162162	194196	125125	G144	1
201	pop16	peach	Kairouan	Spring	27/04/2016	212232	170176	130148	150150	188188	127147	G207	1
202	pop16	peach	Kairouan	Spring	27/04/2016	196238	170172	138148	154154	188194	149151	G27	1

203	pop16	peach	Kairouan	Spring	27/04/2016	212212	170172	138142	154154	188198	153153	G49	1
204	pop16	peach	Kairouan	Spring	27/04/2016	212212	170176	136148	150150	188194	145147	G55	2
205	pop16	peach	Kairouan	Spring	27/04/2016	212212	170176	136148	150150	188194	145147	G55	
206	pop16	peach	Kairouan	Spring	27/04/2016	212238	178178	138142	168168	188194	127127	G295	1
207	pop16	peach	Kairouan	Spring	27/04/2016	212214	170176	138148	150150	188194	153153	G143	1
208	pop16	peach	Kairouan	Spring	27/04/2016	196238	170170	138148	150150	188194	153153	G25	1
209	pop16	peach	Kairouan	Spring	27/04/2016	212212	170172	138142	154154	188198	149149	G48	1
210	pop16	peach	Kairouan	Spring	27/04/2016	212212	176176	138142	154154	188198	149149	G106	1
211	pop16	peach	Kairouan	Spring	27/04/2016	212212	170176	136148	150150	188192	145147	G54	1
212	pop16	peach	Kairouan	Spring	27/04/2016	202204	172178	136136	174176	194194	149149	G34	1
213	pop16	peach	Kairouan	Spring	27/04/2016	212224	170176	138140	156156	188188	145153	G156	1
262	pop17	peach	Kairouan	Winter	14/12/2012	212212	176176	138138	150150	192194	151151	G105	1
280	pop17	peach	Kairouan	Winter	14/12/2012	232240	170178	138142	154154	188192	149151	G375	1
282	pop17	peach	Kairouan	Winter	14/12/2012	212238	172172	138148	168168	188194	151153	G264	1
284	pop17	peach	Kairouan	Winter	14/12/2012	212232	176178	138142	168168	188192	149149	G231	2
285	pop17	peach	Kairouan	Winter	14/12/2012	212232	176178	138142	168168	188192	149149	G231	
286	pop17	peach	Kairouan	Winter	14/12/2012	212232	172176	138142	162162	188192	149149	G221	1
288	pop17	peach	Kairouan	Winter	14/12/2012	212232	170176	138142	168168	188192	149149	G213	1
552	pop17	peach	Kairouan	Winter	14/12/2012	212238	176180	136142	162164	188188	125127	G292	1
553	pop17	peach	Kairouan	Winter	14/12/2012	212236	172176	148148	154156	188198	125151	G240	1
554	pop17	peach	Kairouan	Winter	14/12/2012	212212	170172	136136	150150	188188	153155	G45	1
555	pop17	peach	Kairouan	Winter	14/12/2012	212236	170176	138138	152154	188194	127145	G235	1
556	pop17	peach	Kairouan	Winter	14/12/2012	210212	170172	138142	168168	194194	151153	G39	1
557	pop17	peach	Kairouan	Winter	14/12/2012	212212	170172	142144	152152	194194	153155	G53	1
558	pop17	peach	Kairouan	Winter	14/12/2012	212212	172178	142142	154156	194194	149151	G89	1
559	pop17	peach	Kairouan	Winter	14/12/2012	212212	170172	136138	152152	196196	153155	G47	1

560	pop17	peach	Kairouan	Winter	14/12/2012	212212	170176	138138	154156	190194	127145	G56	1
561	pop17	peach	Kairouan	Winter	14/12/2012	212232	170172	138138	152154	188192	127127	G204	1
562	pop17	peach	Kairouan	Winter	14/12/2012	212236	170178	138142	150152	188192	153155	G237	1
563	pop17	peach	Kairouan	Winter	14/12/2012	212212	172172	138138	150150	188188	153155	G67	1
564	pop17	peach	Kairouan	Winter	14/12/2012	212224	170172	142144	150150	194194	155155	G153	1
565	pop17	peach	Kairouan	Winter	14/12/2012	212224	172176	148148	154156	188188	145149	G174	1
566	pop17	peach	Kairouan	Winter	14/12/2012	212242	172174	136148	152154	188206	125127	G307	1
567	pop17	peach	Kairouan	Winter	14/12/2012	212212	170172	142142	150152	192192	153155	G51	1
568	pop17	peach	Kairouan	Winter	14/12/2012	212238	170176	138148	152154	188190	145149	G262	1
569	pop17	peach	Kairouan	Winter	14/12/2012	212232	170178	138138	152154	188190	127127	G218	1
570	pop17	peach	Kairouan	Winter	14/12/2012	212224	170172	136138	152154	188190	127127	G150	1
571	pop17	peach	Kairouan	Winter	14/12/2012	212224	170172	138138	152152	188204	153155	G151	1
572	pop17	peach	Kairouan	Winter	14/12/2012	196232	170172	142148	154156	188188	127145	G22	1
92	pop18	potato	Kairouan	Winter	05/12/2011	212238	174176	142142	152152	188206	145145	G280	1
93	pop18	potato	Kairouan	Winter	05/12/2011	212224	170176	138148	154154	188194	145155	G159	2
94	pop18	potato	Kairouan	Winter	05/12/2011	212224	170176	138148	154154	188194	145155	G159	
95	pop18	potato	Kairouan	Winter	05/12/2011	212238	172180	142142	172176	188206	145145	G278	1
96	pop18	potato	Kairouan	Winter	05/12/2011	212226	172176	142148	178180	188200	127145	G195	1
369	pop18	potato	Kairouan	Winter	05/12/2011	232240	170176	138142	150152	188194	149151	G366	1
371	pop18	potato	Kairouan	Winter	05/12/2011	232232	178178	138142	150152	192194	149151	G356	1
373	pop18	potato	Kairouan	Winter	05/12/2011	212212	180180	142142	178178	188188	149149	G142	1
374	pop18	potato	Kairouan	Winter	05/12/2011	212224	176178	138142	176178	194202	149151	G184	1
377	pop18	potato	Kairouan	Winter	05/12/2011	210212	174176	138138	150150	188188	149149	G41	1
379	pop18	potato	Kairouan	Winter	05/12/2011	232240	182184	138138	152152	190192	151151	G381	1
380	pop18	potato	Kairouan	Winter	05/12/2011	212222	176178	138142	150152	200200	129129	G148	1
381	pop18	potato	Kairouan	Winter	05/12/2011	232240	176178	138142	150150	194208	151151	G379	1

382	pop18	potato	Kairouan	Winter	05/12/2011	210216	176178	138138	150152	194194	125125	G42	1
383	pop18	potato	Kairouan	Winter	05/12/2011	224224	180180	138138	150152	188192	151151	G317	1
384	pop18	potato	Kairouan	Winter	05/12/2011	212224	170180	138142	150150	188190	147149	G166	1
508	pop18	potato	Kairouan	Winter	05/12/2011	224240	172178	132132	160160	188188	147147	G327	1
509	pop18	potato	Kairouan	Winter	05/12/2011	206208	172172	138142	150150	186188	151153	G35	1
510	pop18	potato	Kairouan	Winter	05/12/2011	212212	178178	142150	152154	194204	127127	G137	1
511	pop18	potato	Kairouan	Winter	05/12/2011	212240	178178	138138	150150	188194	151153	G303	1
512	pop18	potato	Kairouan	Winter	05/12/2011	230240	170180	138142	150150	206208	151153	G352	1
481	pop19	peach	Kairouan	Winter	08/12/2011	212220	172176	138138	160164	188194	127127	G147	1
482	pop19	peach	Kairouan	Winter	08/12/2011	212212	172176	138142	152152	188188	153155	G75	1
483	pop19	peach	Kairouan	Winter	08/12/2011	212212	170178	142150	152152	188194	149151	G65	1
484	pop19	peach	Kairouan	Winter	08/12/2011	210240	172176	142148	152154	188188	127127	G44	1
485	pop19	peach	Kairouan	Winter	08/12/2011	212238	178178	142142	168168	188194	127127	G297	1
486	pop19	peach	Kairouan	Winter	08/12/2011	208224	172178	138138	168168	188188	127127	G37	1
487	pop19	peach	Kairouan	Winter	08/12/2011	212212	170178	136142	150150	188192	127127	G59	1
488	pop19	peach	Kairouan	Winter	08/12/2011	212212	174178	136148	152154	192194	151151	G99	1
489	pop19	peach	Kairouan	Winter	08/12/2011	212238	172178	138148	152154	188188	149151	G274	1
491	pop19	peach	Kairouan	Winter	08/12/2011	212238	170172	138142	154156	188188	127127	G256	1
492	pop19	peach	Kairouan	Winter	08/12/2011	212212	174176	142142	150150	190194	149151	G98	1
494	pop19	peach	Kairouan	Winter	08/12/2011	212212	170178	138138	162162	194210	151153	G62	1
495	pop19	peach	Kairouan	Winter	19/12/2011	232240	170178	138142	150150	194208	151153	G370	3
496	pop19	peach	Kairouan	Winter	19/12/2011	232240	170178	138142	150150	194206	151153	G368	1
497	pop19	peach	Kairouan	Winter	19/12/2011	212224	170172	138142	168168	192194	153155	G152	1
498	pop19	peach	Kairouan	Winter	19/12/2011	212232	170172	138142	150150	206208	151153	G205	1
499	pop19	peach	Kairouan	Winter	19/12/2011	212238	172178	138148	152154	180194	125127	G273	1
500	pop19	peach	Kairouan	Winter	19/12/2011	212224	176178	142142	150150	188194	153155	G185	1

501	pop19	peach	Kairouan	Winter	19/12/2011	232240	170178	138142	150150	194208	151153	G370	
502	pop19	peach	Kairouan	Winter	19/12/2011	232240	170178	138142	150150	194208	151153	G370	
503	pop19	peach	Kairouan	Winter	19/12/2011	212224	170180	138142	154156	194204	149151	G170	1
504	pop19	peach	Kairouan	Winter	19/12/2011	212238	178178	138148	154156	180194	125127	G296	1
505	pop19	peach	Kairouan	Winter	19/12/2011	232240	170178	138142	150150	194208	151151	G369	1
506	pop19	peach	Kairouan	Winter	19/12/2011	232240	170178	138142	152152	194206	149153	G373	1
507	pop19	peach	Kairouan	Winter	19/12/2011	224240	170178	142148	150150	188188	151153	G325	1

Table S2. Polymorphism detected for each microsatellite locus on samples of *Myzus persicae* in Tunisia.

N	548 (Total individuals)				397 (Total MLGs)			
**Locus**	**Na**	**Ho**	**He**	F_{IS}	**Na**	Ho	He	F_{IS}
Myz9	23	0.799	0.763	−0.021 *	23	0.715	0.734	0.026 *
M37	11	0.841	0.789	−0.060 ^{NS}	10	0.803	0.795	−0.012 *
M40	10	0.714	0.737	0.031 *	10	0.647	0.722	0.103 *
M49	20	0.310	0.864	0.641 *	20	0.277	0.870	0.682 *
M63	14	0.712	0.757	0.060 *	14	0.725	0.769	0.057 *
M86	14	0.593	0.848	0.301 *	14	0.559	0.849	0.341 *
Mean	15.333	0.658	0.793	0.158	15.167	0.621	0.790	0.213

* Significant F_{IS} at the level of 5%; ^{NS}: non-significant

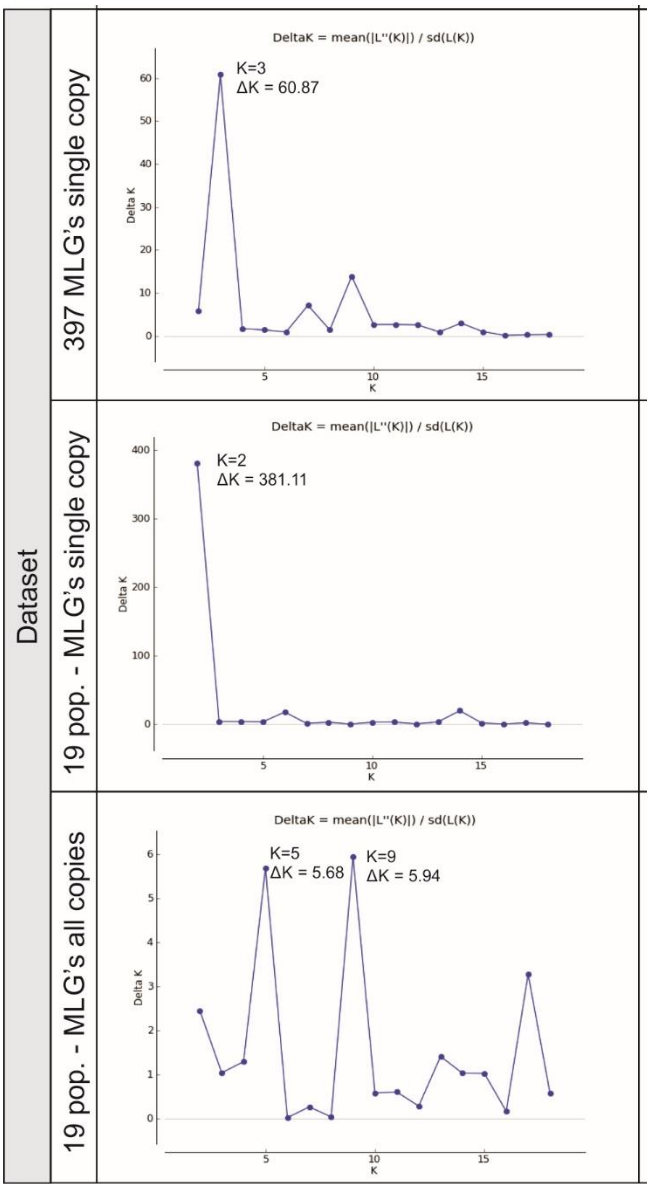

Figure S1. Bayesian population structure analysis used for assigning 397 MLGs and 19 Tunisian populations of *Myzus persicae* to different genetic clusters. The clustering was performed using an admixture model with correlated allele frequencies.
